# Efficacy of telmisartan for the treatment of persistent renal proteinuria in dogs: A double‐masked, randomized clinical trial

**DOI:** 10.1111/jvim.15958

**Published:** 2020-11-09

**Authors:** Bianca N. Lourenço, Amanda E. Coleman, Scott A. Brown, Chad W. Schmiedt, Max C. Parkanzky, Kate E. Creevy

**Affiliations:** ^1^ Department of Small Animal Medicine & Surgery College of Veterinary Medicine University of Georgia Athens Georgia USA; ^2^ Department of Physiology & Pharmacology College of Veterinary Medicine University of Georgia Athens Georgia USA; ^3^ Department of Small Animal Clinical Sciences, College of Veterinary Medicine & Biomedical Sciences, Texas A&M University College Station Texas USA

**Keywords:** angiotensin receptor blocker, angiotensin‐converting enzyme inhibitor, chronic kidney disease, enalapril, renin‐angiotensin‐aldosterone system

## Abstract

**Background:**

Information regarding efficacy of the angiotensin II receptor blocker, telmisartan, for treatment of proteinuria in dogs is limited.

**Objective:**

To evaluate the antiproteinuric efficacy of telmisartan, as compared to enalapril, in dogs with chronic kidney disease and persistent, renal proteinuria.

**Animals:**

Thirty‐nine client‐owned dogs with chronic kidney disease and urinary protein‐to‐creatinine ratio (UPC) > 0.5 (if azotemic) or ≥ 1.0 (if nonazotemic).

**Methods:**

In this prospective, randomized, double‐masked clinical trial, dogs were block randomized, according to presence or absence of azotemia and systemic arterial hypertension, to receive telmisartan (1.0 mg/kg PO q24h), or enalapril (0.5 mg/kg PO q12h), and followed for 120 days. Up‐titration of study drug dosage on days 30 and 60, and addition of the other study drug at day 90, were performed if UPC > 0.5 was noted at these visits. Percentage change in UPC relative to baseline was calculated for all time points. Data are presented as median (range).

**Results:**

Thirty‐nine (20 telmisartan‐treated, 19 enalapril‐treated) dogs were included. At day 30, percentage change in UPC was greater for telmisartan‐treated (−65% [−95% to 104%]) vs enalapril‐treated (−35% [−74% to 87%]) dogs (*P* = .002). Among dogs persistently proteinuric at earlier visits, telmisartan remained superior to enalapril at days 60 (*P* = .02) and 90 (*P* = .02). No difference in percentage change in UPC between study groups was observed at day 120, when combination therapy was allowed. Combination therapy resulted in relevant azotemia in 4/13 (31%) dogs.

**Conclusions and Clinical Importance:**

Telmisartan might be a suitable first‐line therapy for dogs with renal proteinuria.

**Abbreviations**
ACE: angiotensin‐converting enzyme
ACEi: angiotensin‐converting enzyme inhibitor
Ang II: angiotensin II
ARB: angiotensin receptor blocker
CKD: chronic kidney disease
Cr: blood creatinine concentration
Hct: hematocrit
IRIS: International Renal Interest Society
K: blood potassium concentration
RAAS: renin‐angiotensin‐aldosterone system
SBP: systolic arterial blood pressure
UPC: urinary protein‐to‐creatinine ratio
Δ_%_Cr: percentage change in blood creatinine concentration relative to baseline value
Δ_%_Hct: percentage change in hematocrit relative to baseline value
Δ_%_K: percentage change in blood potassium concentration relative to baseline value
Δ_%_UPC: percentage change in urinary protein‐to‐creatinine ratio relative to baseline value

## 1 INTRODUCTION

Chronic kidney disease (CKD) affects up to 1.4% of the general canine population^1^ and 10% of geriatric dogs presented to referral hospitals.^2^ Of these, approximately 52 to 90% are affected by glomerular lesions,^3^, ^4^ of which proteinuria is a hallmark.^5^ In dogs, as in other species,^6^, ^7^ proteinuria is a risk factor for disease progression, and renal and all‐cause mortality.^8^, ^9^, ^10^ Dogs with a urinary protein‐to‐creatinine ratio (UPC) >1.0 are approximately 3 times more likely to experience uremic crises and death than those with UPC ≤1.0.^8^ As interventions that reduce the magnitude of proteinuria are associated with improved outcomes,^11^, ^12^, ^13^ antiproteinuric therapy is considered standard‐of‐care for dogs with proteinuric CKD.^14^, ^15^

Angiotensin‐converting enzyme inhibitors (ACEi), such as enalapril, decrease proteinuria in experimentally‐induced^16^ and naturally‐occurring CKD in dogs.^12^, ^17^ Despite their overall benefit in lowering proteinuria within populations, ACEi are not universally successful, with some dogs experiencing worsening of proteinuria despite therapy.^17^ Further, optimal ACEi dose has not been determined through deliberate dose‐escalation studies in dogs with proteinuric CKD.

Angiotensin II receptor blockers (ARBs) are commonly prescribed to human patients with renal proteinuria.^18^ These drugs reduce urinary protein loss and mitigate progression from microalbuminuria to overt nephropathy.^19^, ^20^, ^21^ All ARBs selectively inhibit the angiotensin II subtype 1 receptor, which mediates the adverse effects of angiotensin II (Ang II) on the cardiovascular system and kidneys.^22^ Selectivity for this receptor subtype provides ARBs a theoretical advantage over ACEi, as the beneficial effects of Ang II binding to Ang II subtype 2 receptors are preserved.^23^ Additionally, ARBs circumvent ACE‐independent proteolytic pathways, which might contribute to persistent Ang II production in patients treated with ACEi.^23^, ^24^

The objective of this study was to determine the short‐term efficacy of telmisartan, compared to a standard dose of enalapril, for the reduction of proteinuria in dogs with persistent pathologic renal proteinuria. We hypothesized that telmisartan would produce a greater percentage reduction in UPC than would enalapril when administered for 30 days. As secondary objectives, we sought to evaluate the efficacy of a dosage‐escalation protocol for dogs in which “standard” dosages of either drug were unsuccessful in controlling proteinuria, and to determine whether treatment with a combination of telmisartan and enalapril for 30 days would lead to a clinically significant UPC reduction in dogs that were persistently proteinuric on “ceiling” dosages of either monotherapy. We hypothesized that telmisartan would lead to a greater and faster reduction in UPC than would enalapril, and that clinically important reductions in UPC would be noted with combination therapy. A final objective of this study was to evaluate the safety of telmisartan and enalapril when administered at progressively greater dosages and when coadministered to a sample of dogs with naturally‐occurring CKD.

## 2 MATERIALS AND METHODS

### 2.1 Study design

This was a prospective, randomized, single‐center, double‐masked clinical trial. All procedures were approved by the Clinical Research Committee of the University of Georgia's College of Veterinary Medicine (approval number CR‐399). Informed owner consent was obtained prior to enrollment.

### 2.2 Animals

Dogs with persistent, pathologic, renal proteinuria due to CKD were recruited prospectively from client‐owned dogs presented to the University of Georgia's Veterinary Teaching Hospital. Dogs of any age and body weight were considered if they had persistent proteinuria with UPC >0.5 if azotemic (ie, blood creatinine [Cr] concentration ≥1.4 mg/dL, IRIS CKD stages 2‐4), or ≥1.0 if nonazotemic (ie, Cr <1.4 mg/dL, IRIS CKD stage 1), documented in ≥2 urine samples collected ≥14 days apart, and abdominal ultrasound findings consistent with CKD.

Dogs were excluded if any 1 or more of the following were identified: urolithiasis; urogenital neoplasia; evidence of hemorrhage, inflammation or bacteria on urine sediment; positive urine culture; positive heartworm antigen test within 3 months of identification of proteinuria or known lack of treatment with regular monthly heartworm preventive; findings suggestive of acute kidney injury, infectious nephropathy or lower urinary tract disease; average indirect systolic arterial blood pressure (SBP) <120 mm Hg; moderate hyperkalemia (blood potassium concentration [K] >6.5 mmol/L); history of having received renin‐angiotensin‐aldosterone system (RAAS) antagonists, corticosteroids, or both, in the 14 days preceding enrollment; and concurrent illness (eg, systemic lupus erythematosus, ehrlichiosis, neoplasia), for which specific treatment might result in mitigation of proteinuria. Dogs with hyperadrenocorticism and diabetes mellitus were not excluded if these diseases were considered clinically and biochemically controlled for at least 30 days prior to enrollment.

### 2.3 Randomization and allocation

Dogs were categorized according to presence or absence of systemic arterial hypertension (ie, average indirect SBP ≥150 mm Hg) and azotemia (ie, Cr ≥1.4 mg/dL). Based on this categorization, dogs were assigned to 1 of 4 groups: nonazotemic, normotensive; nonazotemic, hypertensive; azotemic, normotensive; and azotemic, hypertensive. Within each group, dogs were block randomized in blocks of 4 with a 1 : 1 treatment allocation ratio (telmisartan : enalapril).

### 2.4 Masking

Dog owners, investigators, and study personnel were masked to each dog's treatment group. One of the study investigators (BNL) was unblinded if removal of a dog from the study was necessary for the evaluation or treatment of an adverse event. After completion of each dog's study period, treatment allocation was revealed to 1 of the study investigators (BNL) to enable ongoing treatment recommendations for that dog prior to the conclusion of the study.

### 2.5 Study medications

Dogs were randomized to receive telmisartan solution (Semintra, Boehringer Ingelheim Vetmedica GmbH, Ingelheim, Germany) at an initial dosage of 1.0 mg/kg PO in the morning and an equal volume of placebo in the evening, or enalapril suspension (Enalapril, Taro Pharmaceuticals Industries, Ltd, or Valeant Pharmaceuticals International, Inc, USA, 20 mg tablets compounded into a suspension) at an initial dosage of 0.5 mg/kg q12h PO. Concentrations of telmisartan (10 mg/mL) and enalapril (5 mg/mL) allowed for equivalency of volume administered per kg body weight (0.1 mL/kg) regardless of treatment group. Enalapril was compounded by the UGA Veterinary Teaching Hospital Pharmacy into a suspension using preserved simple syrup and using the standards for compounding provided by the United States Pharmacopeia.^25^ Stability of enalapril in different aqueous suspensions, including deionized water and sweetened suspending agents, is documented.^26^, ^27^, ^28^, ^29^, ^30^, ^31^, ^32^ Further, no clinically relevant differences in pharmacokinetics of commercially available enalapril tablets and compounded liquid formulations prepared from these tablets are observed in children.^33^, ^34^ All study medications were formulated to be visually identical. All owners were provided 2 medication bottles, 1 containing the study drug to be administered in the morning and 1 containing the study drug or placebo to be administered in the evening, to ensure appropriate dosing frequency. Owners were instructed to refrigerate study medication bottles and shake the bottle before administering the medication. Each bottle of medication was used for a maximum period of 35 days. If dogs did not return for reevaluation within that time period, freshly prepared bottles of medication were shipped under refrigeration to their respective owners. For dogs receiving enalapril, new bottles of oral liquid were prepared immediately prior to dispensing the medication. Product variability was minimized by having trained staff prepare the formulation under the oversight and final check of a licensed pharmacist.

### 2.6 Concurrent antihypertensive and nutritional therapies

Dogs receiving amlodipine at the time of enrollment were not excluded. In those dogs for which persistent, severe systolic arterial hypertension (ie, SBP ≥180 mm Hg)^35^ was newly documented at screening, amlodipine was administered at a dosage of 0.1 mg/kg PO q24h alongside the study medication. At rechecks, the dosage of amlodipine was adjusted at the clinician's discretion, to a maximum dosage of 0.3 mg/kg q12h, targeting SBP between 100 and 180 mm Hg. Unless contraindicated, dogs were maintained on a commercially available renal diet or a homemade diet formulated by a certified veterinary nutritionist to be low in phosphorus and protein, alongside a polyunsaturated fatty acid supplement. Any nutritional interventions were initiated at least 14 days prior to study enrollment, and no changes to diet were allowed during the study period.

### 2.7 Schedule of events

**FIGURE 1:**
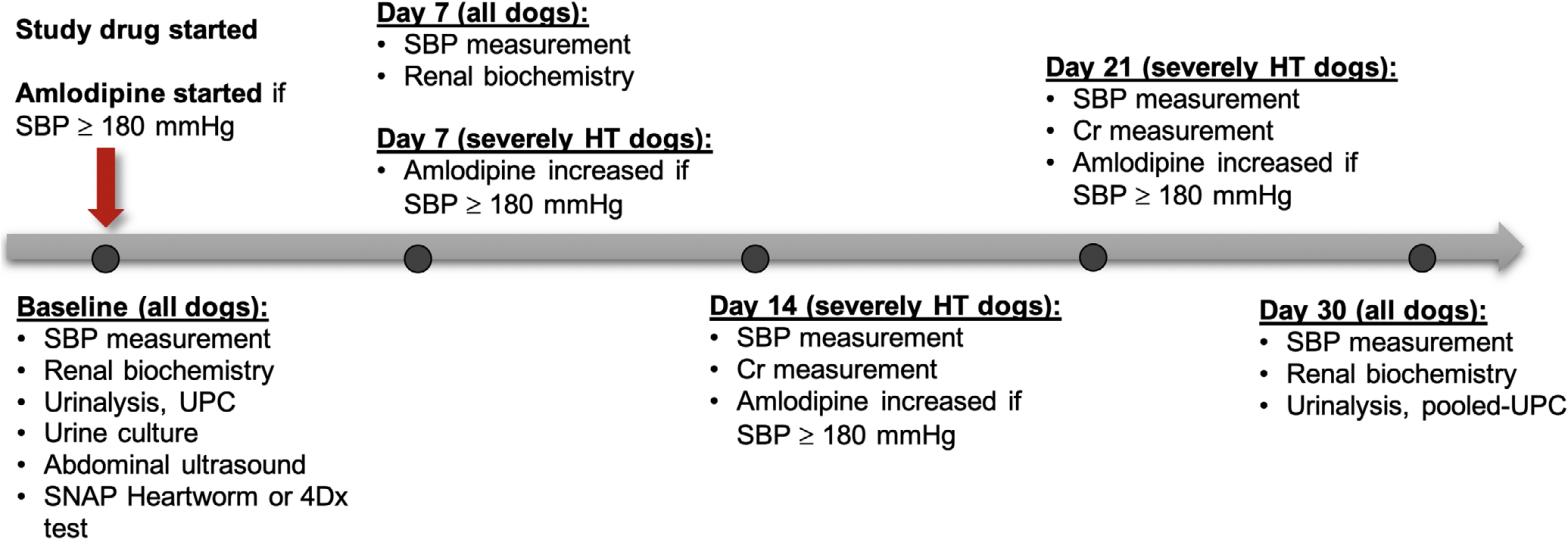
Overview of study phase I. Cr, blood creatinine concentration; HT, hypertensive; SBP, systolic blood pressure in mm Hg; UPC, urinary protein‐to‐creatinine ratio

**FIGURE 2:**
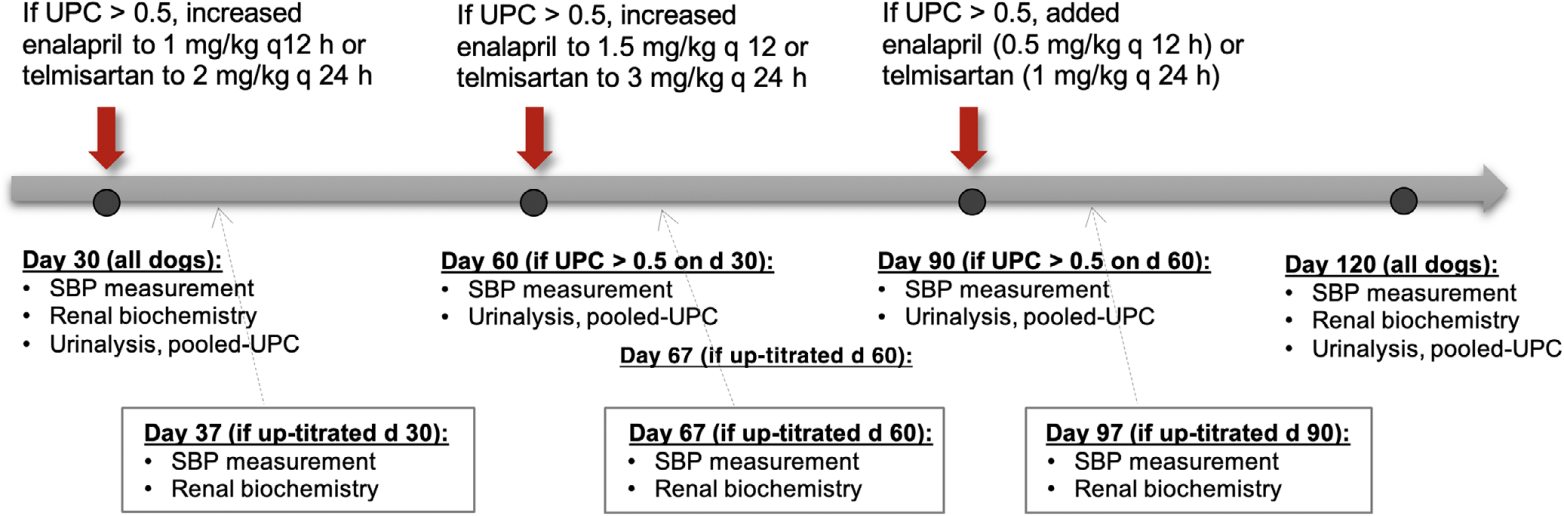
Overview of study phase II. HT, hypertensive; SBP, systolic blood pressure in mm Hg; UPC, urinary protein‐to‐creatinine ratio

This study consisted of 2 phases. Phase I was a 30‐day period during which dogs received telmisartan or enalapril at “standard” doses. Phase II was a subsequent 90‐day period during which monthly up‐titration of study drug dosage and subsequent addition of the other study drug was performed step‐wise to target UPC ≤0.5 in persistently proteinuric dogs. All dogs were reevaluated at the end of the 120‐day study period. The monitoring protocol used in the present study was adapted from recommendations of the American College of Veterinary Internal Medicine.^15^ General trial design is outlined in Figures 1 and 2.

At enrollment (day 0), physical examination data, indirect SBP, and urine for UPC measurement were obtained for all dogs. In addition, blood for measurement of hematocrit (Hct), serum biochemical analyses, as well as *Dirofilaria immitis* antigen test (SNAP Heartworm RT Test, IDEXX Laboratories, Westbrook, Maine) or a combined test for *Anaplasma phagocytophilum*, *Anaplasma platys*, *Borrelia burgdorferi*, and *Ehrlichia canis* and *Ehrlichia ewingii* antibodies and *D. immitis* antigen (SNAP 4Dx Plus, IDEXX Laboratories, Westbrook, Maine), and urine obtained by cystocentesis for urinalysis and urine culture, were collected if these data had not been obtained in the 14 days prior to enrollment. In addition, whole blood biochemical analyses (Stat Profile pHOx Ultra, Nova Biomedical Corporation, Waltham, Massachusetts) for variables indicative of renal function was performed to provide baseline values that would be directly comparable to follow‐up values. Dogs underwent abdominal ultrasonographic examination, performed by a board‐certified veterinary radiologist or a trainee under the supervision of a radiologist, if such an examination had not been performed within the 8 weeks preceding enrollment. Endocrine disease testing was performed if clinical signs or bloodwork findings were suspicious for hyperadrenocorticism or hypothyroidism. All laboratory analyses were performed by a single laboratory (The University of Georgia's Veterinary Teaching Hospital Clinical Pathology Laboratory, Athens, Georgia).

Systolic blood pressure was measured at all visits using Doppler ultrasonography (Model 811‐B Doppler Ultrasonic Flow Detector, Parks Medical Electronics, Inc, Aloha, Oregon), following an acclimation period of at least 10 minutes and before physical examination, venipuncture or cystocentesis, in a manner conforming to guidelines set forth by the American College of Veterinary Internal Medicine.^35^ For each measurement session, after the first measurement was discarded, 5 consecutive consistent measurements were recorded, and the average of these used as the SBP value for that session. Baseline UPC was defined as the average of 2 measurements, 1 obtained on study day 0, and the other obtained within the 30 days preceding enrollment in the absence of an active urinary sediment, urinary tract infection, or treatment with RAAS antagonists or corticosteroids. If UPC measurement was not performed within the 30 days preceding enrollment or did not meet the above criteria, UPC at study day 0 was used as the baseline value. For all subsequent study timepoints, UPC was determined from a pooled urine sample, created by combining equal aliquots from 3 voided samples collected by owners on 3 mornings preceding the visit.^36^

### 2.8 Study phase I

Scheduled rechecks were performed on days 7 ± 1 and 30 ± 2 for all dogs, at which time physical examination data, SBP, and whole blood for renal biochemical analyses were obtained. Urinalysis and UPC measurement were repeated on day 30 ± 2.

In hypertensive dogs for which SBP ≥180 mm Hg was documented at day 7 ± 1, amlodipine was increased to 0.1 mg/kg PO q12h, and SBP and Cr rechecked at 7‐day intervals. At follow‐up visits, amlodipine dosage was increased in increments of 0.05 mg/kg q12h to a maximum dose of 0.3 mg/kg q12h, to target SBP <180 mm Hg.

Dogs were removed from the study if an increase in Cr of ≥30% compared to baseline or moderate hyperkalemia was identified at any recheck, or if hypotension (ie, SBP <100 mm Hg with compatible clinical signs) was identified in a dog not receiving amlodipine. Dogs receiving amlodipine could remain in the study if dosage decrease or discontinuation led to resolution of hypotension. Study medication dosage decreases were not permitted.

### 2.9 Study phase II

Dogs with UPC ≤0.5 at day 30 ± 2 continued to receive study drug at the originally prescribed dosage and were not reevaluated until the end of the study (day 120 ± 2). For dogs with UPC >0.5 at day 30 ± 2, study drug dosage was increased to 2.0 mg telmisartan/kg q24h or 1.0 mg enalapril/kg q12h according to treatment group. At day 60 ± 2, UPC was reevaluated in dogs undergoing dosage up‐titration at day 30 and dogs either continued to receive study drug at the same dosage until final study recheck on study day 120 (those with UPC ≤0.5), or underwent dosage up‐titration to 3.0 mg telmisartan/kg q24h or 1.5 mg enalapril/kg q12h (those with UPC >0.5). At day 90 ± 2, UPC was reevaluated in dogs undergoing dosage up‐titration at day 60, and dogs either continued to receive study drug at the same dosage either alone (those with UPC ≤0.5), or additionally received “standard” dose of the other study drug (1.0 mg telmisartan/kg q24h or 0.5 mg enalapril/kg q12h; those with UPC > 0.5) until the final study recheck.

Dogs undergoing study drug up‐titration or addition of the other study drug were rechecked 7 days after each treatment adjustment (ie, on days 37 ± 1, 67 ± 1, and/or 97 ± 1), at which time physical examination data, SBP, and whole blood for biochemical analyses for variables indicative of renal function were obtained. Removal criteria for phase II were identical to those of phase I. As noted, any dog with UPC ≤0.5 at a scheduled recheck was not reevaluated until study end.

All dogs underwent reevaluation on day 120 ± 2, at which time physical examination data, SBP, blood for measurement of Hct and biochemical analyses, and urine for urinalysis and UPC measurement were obtained.

### 2.10 Study samples considered

Dogs that were randomized and received at least 1 dose of study medication comprised the intention‐to‐treat sample. Dogs that were confirmed to have met all eligibility criteria comprised the per‐protocol sample.

### 2.11 Outcome variables

Primary outcome variables related to efficacy were percentage change in UPC compared to baseline (Δ_%_UPC), calculated by subtracting baseline UPC from recheck UPC and dividing the difference by baseline UPC, at each of days 30 ± 2, 60 ± 2, 90 ± 2, and 120 ± 2. Additional efficacy outcomes included Δ_%_UPC at the maximum‐tolerated study drug dosage (ie, the maximum tested dosage at which no adverse events triggering removal from the study were experienced); the proportion of dogs and odds of achieving UPC reduction ≥50%, and proportion and odds of dogs achieving UPC ≤0.5 at each of days 30 ± 2, 60 ± 2, 90 ± 2, and 120 ± 2; as well as time‐to‐UPC reduction ≥50% and time‐to‐UPC ≤0.5.

Safety outcomes of interest included percentage change from baseline in Cr (Δ_%_Cr), K (Δ_%_K), Hct (Δ_%_Hct) and SBP (Δ_%_SBP) at each of days 30 ± 2, 37 ± 1, 67 ± 1, 97 ± 1, and 120 ± 2, calculated in the same manner as Δ_%_UPC, as well as removal and adverse events. An adverse event was defined as any unfavorable or unintended observation recorded during the study.

### 2.12 Statistical analyses

#### 2.12.1 Power calculation

Due to expected interday variability in UPC, serial measurements must differ by >40% to confidently attribute any observed reduction to a given intervention.^14^ Therefore, UPC reduction ≥50% was considered clinically relevant in the present study. Prior work has demonstrated a mean UPC reduction of 51% in proteinuric dogs treated with enalapril.^14^ Based on this assumption, 27 dogs per treatment group were considered necessary to identify UPC reduction ≥50% with a statistical power of 80% and 5% alpha error level, utilizing a 2‐tailed test.

#### 2.12.2 Interim monitoring

Interim analysis was scheduled for January 2019, the expected end of the study period. It was determined a priori that the trial would be terminated if a statistically significant difference in Δ_%_UPC was identified between treatment groups at day 30, and if median UPC reduction from baseline ≥50% was noted in at least 1 treatment group at day 30. A uniform alpha‐spending function was used to determine significance level (*P* = .01), with the number of enrolled dogs at the time of analysis used as the information fraction (t*).^37^ Interim analyses were conducted by an independent statistician, who did not participate in case recruitment, management or follow‐up.

#### 2.12.3 Final study analyses

Statistical analyses were performed using commercially available software packages (R Development Core Team, version 3.6.1, Vienna, Austria, and GraphPad Prism for Mac, version 8.3.0, GraphPad Software, Inc, La Jolla, California). A significance level of 0.05 was used for all analyses. Data were examined for normality by visual assessment of histograms and normal quantile plot, and the Shapiro‐Wilk test. Normally distributed data are presented as mean ± SD and compared between groups using the Student's *t* test. Nonnormally distributed data are presented as median (range) and compared using the Wilcoxon rank sum test. Proportion of dogs and odds of achieving a given endpoint were compared using the Fisher's exact test. Time‐to‐event analyses were performed using the Kaplan Meier estimator function and the log‐rank test.

To test the influence of magnitude of baseline proteinuria on response to therapy, a stratified, nonparametric permutation test was performed on Δ_%_UPC at day 30, controlling for baseline UPC. Four strata were created based on baseline UPC quartiles (<1.623, 1.623‐3.82, 3.83‐5.78, and > 5.78). An alternative model, using arbitrarily defined cut points (<2.00, 2.0‐4.99, 5.00‐7.00, and > 7.0) was also tested. The permutation test was performed by permuting the class labels within each stratum, recalculating the Wilcoxon rank sum test statistic, and comparing the test statistic for the null hypothesis of no difference between the treatment groups on the original data to the distribution on the stratification‐permuted datasets.

All analyses were performed on the intention‐to‐treat sample, meaning that the baseline randomization was used for primary evaluation of treatments, and treatment modification information was not used for primary comparisons. Data from dogs not evaluated at a given time point according to study protocol or due to removal from the study were treated as missing. Select efficacy outcome variables were also compared in the per‐protocol sample, and are reported as such.

## 3 RESULTS

Recruitment, enrollment and follow‐up were carried out from January 1, 2015 to April 4, 2019.

Planned interim analysis, performed using data collected through December 31, 2018 from 39 dogs for which data from the first 30 days (Phase I) of study were available, was conducted in January 2019, at which time criteria for trial termination were met and enrollment was ceased. Six dogs that were actively enrolled at the time of interim analysis were followed to the end of phase II in a double‐masked manner. For these 6 dogs, UPC reduction relative to baseline ≥50% and UPC ≤0.5 were noted in n = 6 and n = 4, respectively. Investigators remained masked to individual drug assignments, as well as to the identity of the superior drug, until all dogs completed the entire study period.

**FIGURE 3:**
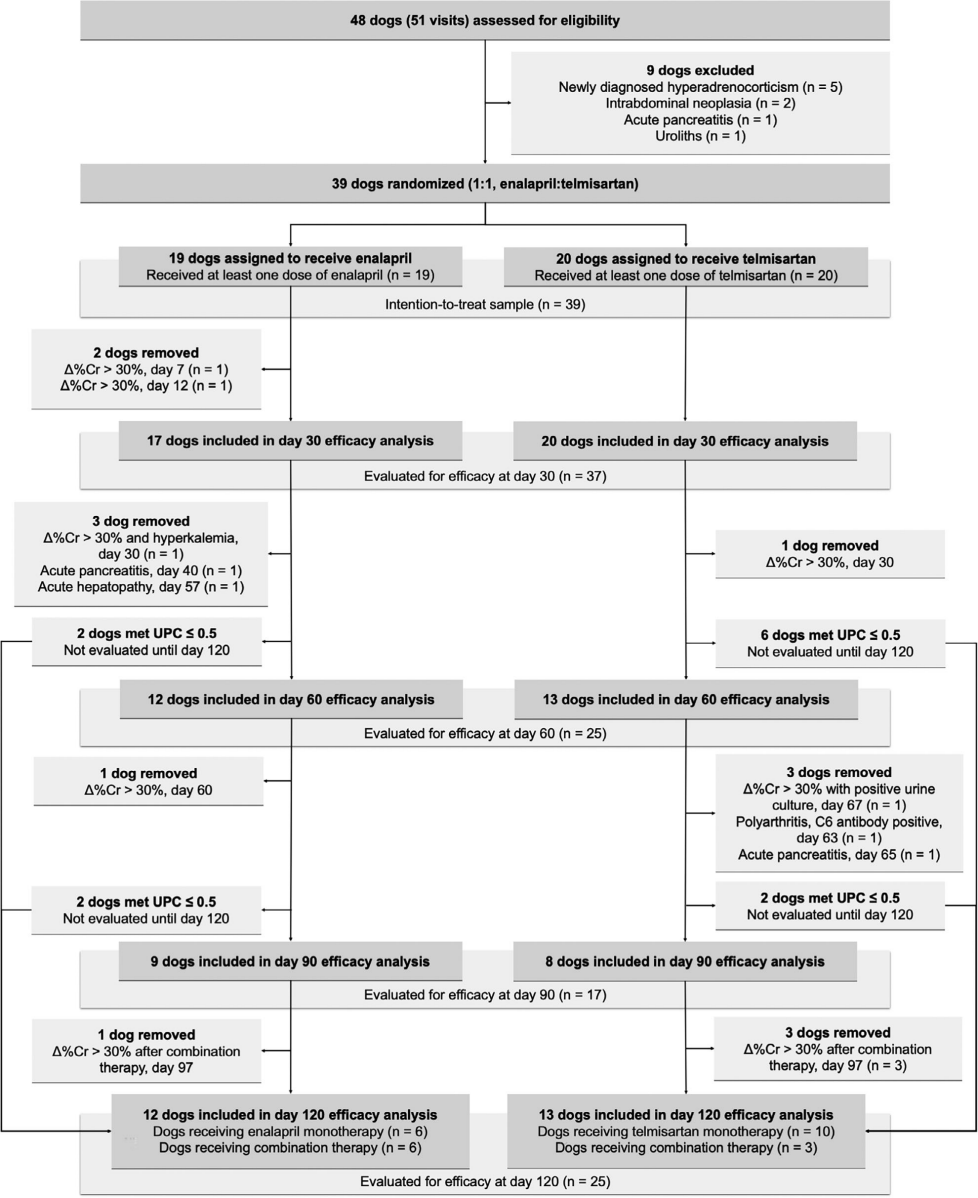
Flow diagram illustrating the progress of dogs through the present study

A total of 48 dogs were screened for eligibility, of which 39 were included in the intention‐to‐treat sample and were randomized to receive enalapril (n = 19) or telmisartan (n = 20; Figure 3). One telmisartan‐treated dog, included in the intention‐to‐treat sample, was discovered after enrollment to be infected with *B. burgdorferi* (ie, a systemic infection for which specific treatment might result in mitigation of proteinuria) and therefore excluded from the per‐protocol sample, leaving 38 dogs in the latter.

Baseline characteristics were similar between the 2 treatment groups (Table 1). The majority of included dogs were nonazotemic (74%) and hypertension was documented in most (62%). Three telmisartan‐ and 2 enalapril‐treated dogs were severely hypertensive at baseline. Historical or comorbid diseases were reported in all but 2 enalapril‐ and 1 telmisartan‐treated dogs (Table 2). Most dogs were receiving a renal diet (82%), fish oil supplementation (87%), or both (Table 3). One dog in each treatment group was receiving amlodipine at enrollment.

**TABLE 1:** Baseline demographic, clinical, and clinicopathologic data for 39 dogs comprising the intention‐to‐treat population

Variable	Enalapril group	Telmisartan group
Number in the intention‐to‐treat	19	20
Age (years)	10.4 (3.1‐14.5)	8.8 (4.3‐14.9)
Sex (n)		
Female spayed	13	14
Male neutered	6	6
Body weight (kg)	13.4 (3.5‐41.3)	11.5 (4‐42.8)
Breed (n)		
Jack Russell terrier	4	0
Beagle	2	2
Miniature Schnauzer	0	2
Boston terrier	0	2
Golden retriever	2	0
Fox terrier	0	2
Yorkshire terrier	2	0
Other (n < 2)	8	9
Mixed breed	1	3
Systolic arterial blood pressure (mm Hg)	154 (126‐210)	154 (120‐220)
Blood creatinine concentration (mg/dL)	0.9 (0.5‐5.5)	0.9 (0.7‐5.0)
Blood urea nitrogen concentration (mg/dL)^a^	11 (8‐101)	13 (5‐64)
Blood potassium concentration (mmol/L)	4.32 (3.89‐5.5)	4.39 (3.88‐4.88)
Serum albumin concentration (g/dL)^b^	3.4 (1.7‐3.9)	3.3 (2.2‐3.9)
Hematocrit (%)	45 (35‐53)	46 (29‐53)
Urinary protein‐to‐creatinine ratio^c^	2.29 (0.91‐15.54)	4.65 (0.90‐13.39)
Study group (n)		
Nonazotemic, normotensive	6	6
Nonazotemic, hypertensive	9	8
Azotemic, normotensive	1	2
Azotemic, hypertensive	3	4
IRIS CKD stage (n)		
1	15	14
2	1	3
3	2	3
4	1	0

*Note*: Data are presented as median (range) where appropriate. azotemic, blood creatinine concentration (Cr) ≥1.4 mg/dL; hypertensive, average systolic blood pressure ≥150 mm Hg.

Abbreviations: CKD, chronic kidney disease; IRIS, International Renal Interest Society.

a For one dog, blood urea nitrogen concentration was greater than the upper limit of reporting for the assay (100 mg/dL) and was assigned a value of 101 mg/dL.

b Baseline albumin was measured in only n = 17 enalapril‐ and n = 14 telmisartan‐treated dogs.

c Baseline urinary protein‐to‐creatinine ratio (UPC) is presented as the average of two measurements, one obtained on study day 0, and the other obtained within the 30 days preceding enrollment for n = 10 enalapril‐ and n = 9 telmisartan‐treated dogs. For all other dogs, the UPC measured at day 0 is used for the baseline value.

**TABLE 2:** Known or reported historical and concurrent conditions in 39 dogs comprising the intention‐to‐treat sample

		Enalapril group	Telmisartan group
	Total number	19	20
Concurrent conditions	Orthopedic disease	5	6
	Periodontal disease	5	5
	ACVIM stage B1 MMVD	3	2
	Heart murmur with open diagnosis	2	3
	Hypothyroidism	2	2
	Atypical hyperadrenocorticism	0	1
	Urinary incontinence	2	2
	Clitoral hypertrophy	0	1
	Suspect renal dysplasia	0	1
	Chronic pancreatitis	0	3
	Food‐responsive gastroenteropathy	1	0
	Stress colitis	0	1
	Ocular disease	2	3
	Chronic respiratory disease	2	1
	Nodular hepatopathy	3	1
	Gall bladder mucocele	1	0
	Atopic dermatitis	1	2
	Aural hematoma	1	0
	Acral lick granuloma	0	1
Historical conditions	Completely excised neoplasia	5	3
	Subcutaneoous lipomas	3	1
	Hepatosplenic infarction	0	1
	Acute pancreatitis	1	0
	Intervertebral disc disease	1	0
	Immune‐mediated thrombocytopenia	0	1
	Esophageal foreign body	1	0
	None reported	2	1

*Note*: Data are presented as number of dogs.

Abbreviation: MMVD, myxomatous mitral valve disease.

**TABLE 3:** Concurrent medications, nutritional therapy and polyunsaturated fatty acid supplementation in 39 dogs comprising the intention‐to‐treat population

		Enalapril group	Telmisartan group
	Total number	19	20
Clinical renal diet	Yes	17 (89%)	15 (75%)
	No	2 (11%)	5 (25%)
Polyunsaturated fatty acid supplementation	Yes	18 (95%)	16 (80%)
	No	1 (5%)	4 (20%)
Concurrent oral medications	Levothyroxine	2 (11%)	2 (10%)
	Trilostane	0	1 (5%)
	Phenylpropanolamine	1 (5%)	2 (10%)
	Ursodeoxycholic acid	2 (11%)	1 (5%)
	S‐Adenosylmethionine + silybin	0	2 (10%)
	Pimobendan	1 (5%)	1 (5%)
	Amlodipine^a^	6 (32%)	4 (20%)
	Gabapentin	0	1 (5%)
	Tramadol	3 (16%)	0
	Trazodone	1 (5%)	1 (5%)
	Amantadine	0	1 (5%)
	Carprofen	0	1 (5%)
	Clopidogrel	0	1 (5%)
	Maropitant	0	1 (5%)
	Famotidine	1 (5%)	0
	Metoclopramide	0	1 (5%)
	Metronidazole	0	1 (5%)
	Theophylline	0	1 (5%)
	Hydrocodone	0	1 (5%)
	Diphenhydramine	0	1 (5%)
Ophthalmic ointments	Cyclosporine	1 (5%)	0
	Neomycin/Polymixin B/Gramicidin	1 (5%)	0
	Neomycin/Polymixin B	1 (5%)	0
Injectable	Canine Atopic Dermatitis Immune IL‐31 monthly	0	1 (5%)

*Note*: Concurrent medications are reported if administered at least once at any point during the study. Data are presented as number of dogs (%).

a One dog in each group was receiving amlodipine prior to study enrollment. In the remainder, amlodipine was added as part of study protocol.

A total of 25 (13 telmisartan‐treated and 12 enalapril‐treated) dogs completed the 120‐day study period (Figure 3). Five dogs in each group were removed after measured concentrations of Cr, K, or both, triggered removal according to study protocol. Three dogs were removed after developing an illness that required investigator unmasking for clinical decision‐making. One telmisartan‐treated dog was removed from the study on day 63 after developing progressive clinical signs (including polyarthropathy) and diagnostic testing results (eg, joint fluid cytology, positive C6 antibody test) consistent with *B. burgdorferi* infection. It was discovered that the dog had known tick exposure during travel to Maine 1 week prior to study inclusion. Data from this dog and all other removed dogs are included in the intention‐to‐treat analyses at all time points prior to removal; however, this dog was not included in the per‐protocol sample.

### 3.1 Protocol adherence

Relevant deviations from study protocol occurred in 4 cases. In 3 (2 telmisartan‐ and 1 enalapril‐treated), a deviation occurred when the prospect of protocol‐dictated dosage adjustments or additions raised safety concerns in a patient experiencing a plateau in Δ_%_UPC and progressive Cr increase following previous up‐titrations. Such deviations occurred at study day 60 in 1 telmisartan‐treated dog, for which up‐titration was not performed (this dog was subsequently removed on day 65 after developing acute pancreatitis) and at study day 90 in the 2 remaining dogs, from which the second study drug was withheld. Three days after initiation of combination therapy (day 93), the owner of the fourth dog discontinued both study drugs; this dog was subsequently removed on day 97 for progressive azotemia. Two of the 4 dogs with known protocol deviations were removed prior to the next scheduled UPC measurement. For the 2 remaining dogs, 1 in each treatment group, because these deviations resulted in withholding of the second study drug, any potential impact would be limited to day 120 data.

### 3.2 Antihypertensive therapy

Ten dogs (n = 6 in enalapril group, n = 4 in telmisartan group) were treated with amlodipine during the study. Median (range) dosage of amlodipine administered at each dog's final recheck was 0.21 (0.09‐0.35) mg/kg/day and 0.13 (0.09‐0.35) mg/kg/day for the enalapril and telmisartan groups, respectively. Median dosage of amlodipine at final recheck was not significantly different between groups (*P* = .39).

### 3.3 Phase I efficacy

**FIGURE 4:**
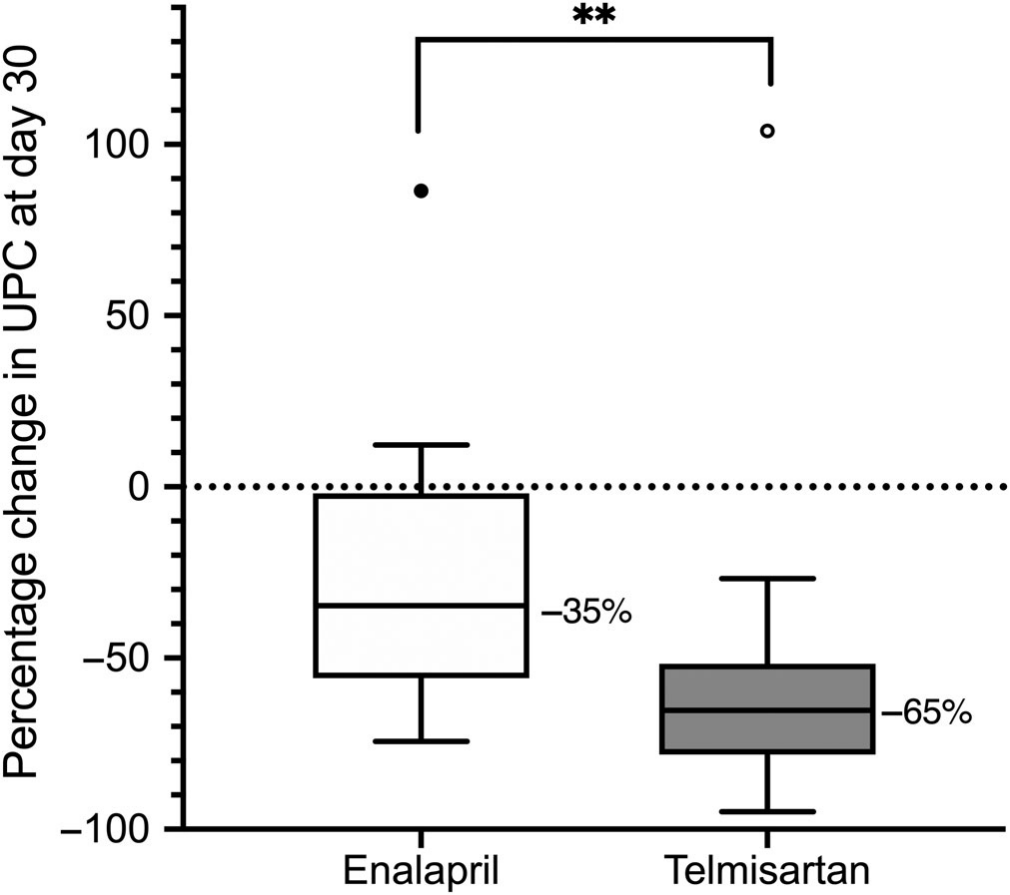
Box‐plot of percentage change in urinary protein‐to‐creatinine ratio (UPC) relative to baseline after 30 days of therapy in 17 dogs receiving enalapril (0.5 mg/kg PO q12h) and 20 dogs receiving telmisartan (1 mg/kg PO q24h). Boxes represent interquartile range, and the horizontal bar within each box and numbers to the right of it represent the median. Upper and lower bars and outliers (closed circles) are plotted using the method of Tukey. One telmisartan‐treated dog, represented by the open outlier data point, was later found to have an active *Borrelia burgdorferi* infection. **P < .01

At day 30, telmisartan‐treated dogs experienced significantly greater median reduction in proteinuria than did enalapril‐treated dogs (*P* = .002; Figure 4); this difference was also significant in the per‐protocol sample (*P* < .001). When controlled for baseline UPC, the difference in Δ_%_UPC remained significant, regardless of the cut‐points applied (stratified permutation *P* < .001 and *P =* .001 for quartile and alternative cut‐points, respectively).

A greater proportion of telmisartan‐ vs enalapril‐treated dogs experienced UPC reduction relative to baseline ≥50% on day 30 (Table 4). The odds of achieving this endpoint by day 30 were 6.9 times higher in telmisartan‐ compared to enalapril‐treated dogs. There was no significant difference in the proportion of dogs with UPC ≤0.5 at day 30 in each treatment group (Table 5).

**TABLE 4:** Proportion of proteinuric dogs achieving reduction in urinary protein‐to‐creatinine ratio (UPC) ≥50% in response to treatment with PO administered enalapril or telmisartan at progressively greater dosages to target UPC ≤0.5

	Proportion of dogs achieving a ≥50% reduction in UPC		Odds ratio	*P*‐value
	Enalapril	Telmisartan		
Day 30	6/17 (35%)	16/20 (80%)	6.9	.008
Day 60	5/12 (42%)	10/13 (77%)	4.4	.11
Day 90	4/9 (44%)	7/8 (88%)	7.6	.13
Day 120	12/12 (100%)	12/13 (92%)	0	1
Monotherapy (various dosages)	6/6 (100%)	9/10 (90%)		
Second drug added	6/6 (100%)	3/3 (100%)		
At maximum‐tolerated dosage	8/16 (50%)	16/19 (84%)	5.1	.06

*Note*: Dogs achieving UPC ≤0.5 at earlier time points were not reevaluated until day 120. Data are presented as number of dogs achieving the endpoint/number of dogs examined at a given timepoint.

**TABLE 5:** Proportion of proteinuric dogs achieving urinary protein‐to‐creatinine ratio (UPC) ≤ 0.5 in response to treatment with PO administered enalapril or telmisartan at progressively greater dosages to target this outcome

	Proportion of dogs achieving UPC ≤0.5		Odds ratio	*P*‐value
	Enalapril group	Telmisartan group		
Day 30	2/17 (12%)	6/20 (30%)	3.2	.25
Day 60	2/12 (17%)	2/13 (15%)	0.9	1
Day 90	1/9 (11%)	7/8 (13%)	1.1	1
Day 120	8/12 (67%)	9/13 (69%)	1.1	1
Monotherapy (various dosages)	5/6 (83%)	8/10 (80%)		
Second drug added	3/6 (50%)	1/3 (33%)		
Maximum‐tolerated dosage	5/16 (31%)	9/19 (47%)	2.0	.47

*Note*: Dogs achieving UPC ≤0.5 at earlier time points were not reevaluated until day 120. Data are presented as number of dogs achieving the endpoint/number of dogs examined at a given timepoint.

### 3.4 Phase II efficacy

**FIGURE 5:**
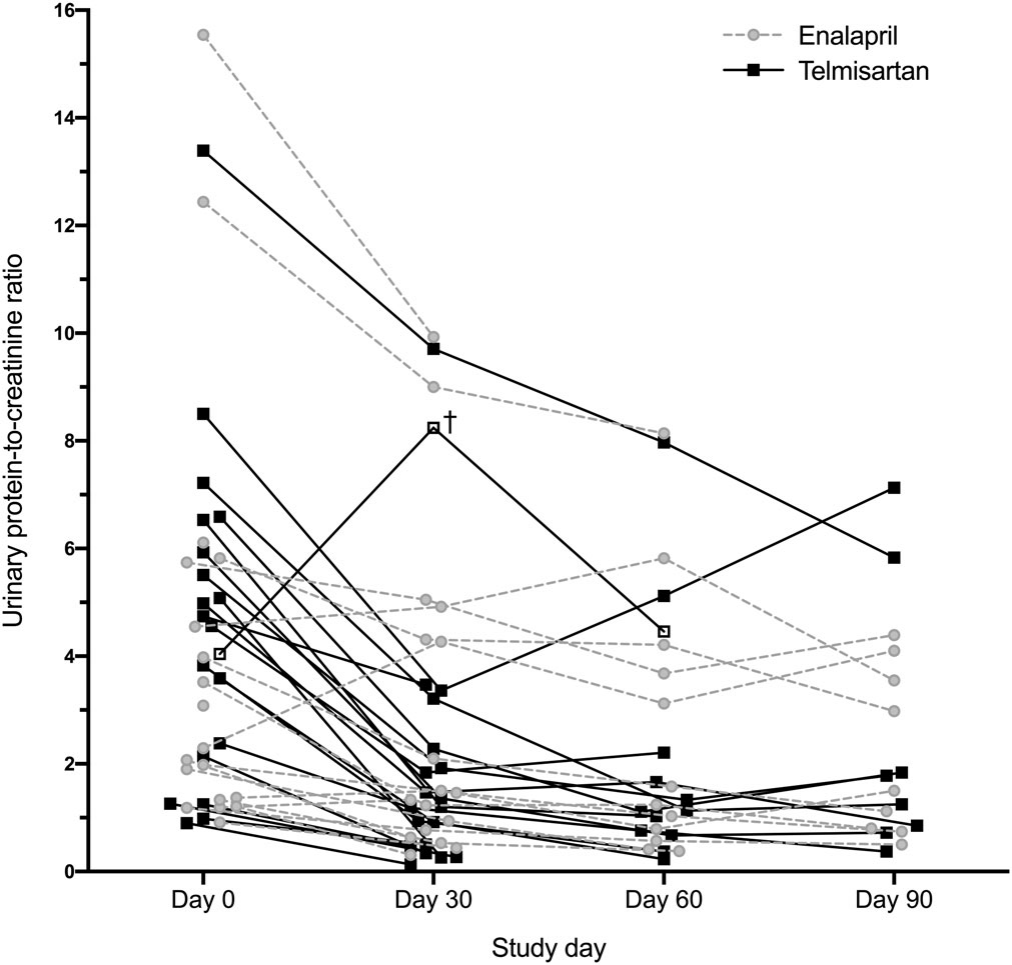
Urinary protein‐to‐creatinine ratio (UPC) in proteinuric dogs administered enalapril (0.5‐1.5 mg/kg PO q12h) or telmisartan (1‐3 mg/kg PO q24h) at progressively greater dosages to target UPC ≤0.5. Administration of study drugs was initiated at day 0, and UPC was measured every 30 days until UPC ≤0.5 was documented or dog was removed from the study for azotemia, hyperkalemia, hypotension, or a combination of these. Lines connect measurements from a given individual. †, Dog later found to have *Borrelia burgdorferi* infection

**FIGURE 6:**
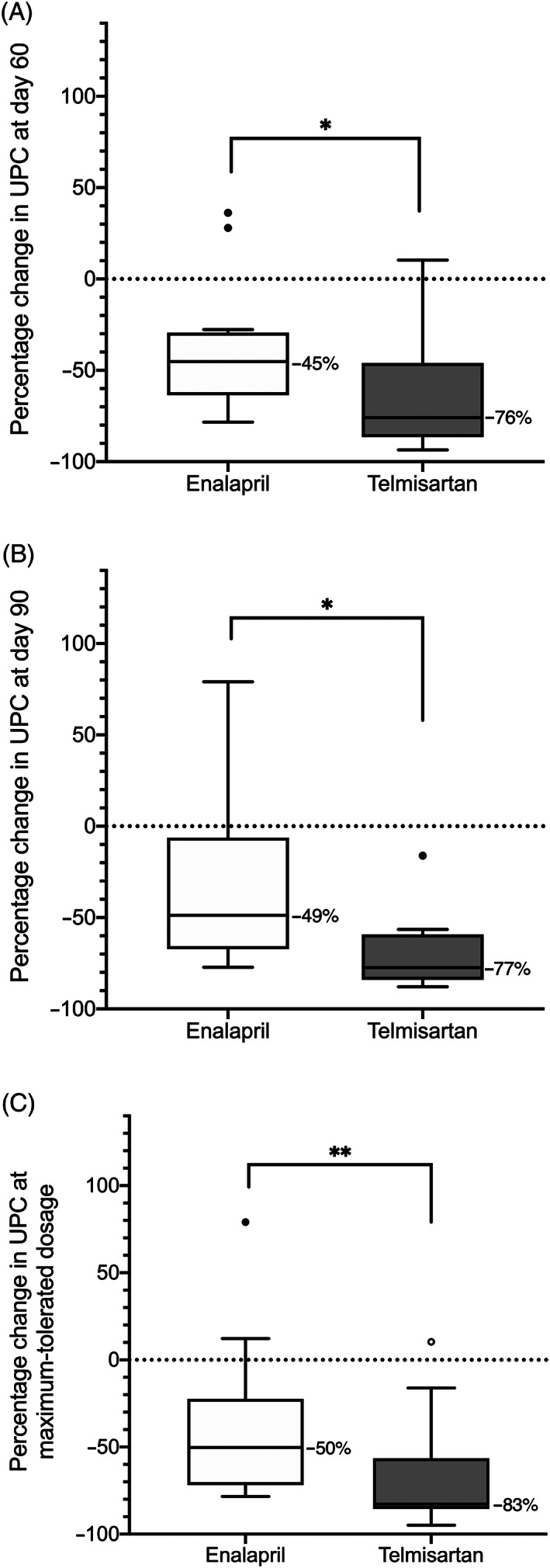
Box‐plot of percentage change in urinary protein‐to‐creatinine ratio (UPC) relative to baseline in proteinuric dogs randomized to receive enalapril or telmisartan. A, Percentage change in UPC relative to baseline after 60 days of therapy in 12 dogs receiving enalapril (0.5 mg/kg PO q12h for 30 days, followed by 1.0 mg/kg PO q12h thereafter) and 13 dogs receiving telmisartan (1 mg/kg PO q24h for 30 days, followed by 2 mg/kg PO q24h thereafter). B, Percentage change in UPC relative to baseline after 90 days of therapy in 9 dogs receiving progressively greater dosages of enalapril (0.5 mg/kg PO q12h on study days 0‐30, 1.0 mg/kg PO q12h on study days 31‐60, 1.5 mg/g PO q12h on study days 61‐90) and 8 dogs receiving progressively greater dosages of telmisartan (1 mg/kg PO q24h on study days 0‐30, 2 mg/kg PO q24h on study days 31‐60, 3 mg/kg PO q24h on study days 61‐90). C, Percentage change in UPC relative to baseline in 16 dogs receiving the maximum‐tolerated dosage of enalapril (ranging from 0.5‐1.5 mg/kg PO q12h) and 19 dogs receiving the maximum‐tolerated dosage of telmisartan (ranging from 1‐3 mg/kg PO q24h). Maximum‐tolerated dosage for a given dog was defined as the maximum dosage received without the occurrence of adverse events that would trigger removal from the study (ie, hypotension, azotemia, hyperkalemia, or any combination of these). Boxes represent interquartile range, and the horizontal bar within each box and numbers to the right of it represent the median. Upper and lower bars and outliers (circles) are plotted using the method of Tukey. One telmisartan‐treated dog, represented by the open outlier data point, was later found to have an active *Borrelia burgdorferi* infection. *P < .05. **P < .01

Values for UPC at baseline and on study days 30, 60, and 90 are presented in Figure 5. In dogs with proteinuria refractory to standard dosages of study drug, greater median reduction in UPC was noted for those treated with progressively greater dosages of telmisartan than for those treated with progressively greater dosages of enalapril at days 60 (*P* = .02) and 90 (*P* = .02; Figure 6). The difference between treatment groups was also significant in the per‐protocol sample (*P* < .001 at day 60).

To assess drug efficacy while considering clinical usefulness of the tested protocols, Δ_%_UPC at maximum‐tolerated tested dosage was compared between groups. Dogs receiving telmisartan to target UPC ≤0.5 experienced significantly median greater reduction in UPC than those receiving enalapril for the same purpose (*P* = .004; Figure 6). There was no significant difference in the proportion of dogs or odds of achieving the clinical goals of UPC reduction from baseline ≥50% (Table 4) or UPC ≤0.5 (Table 5) at days 60 or 90, or at maximum‐tolerated dosages.

**FIGURE 7:**
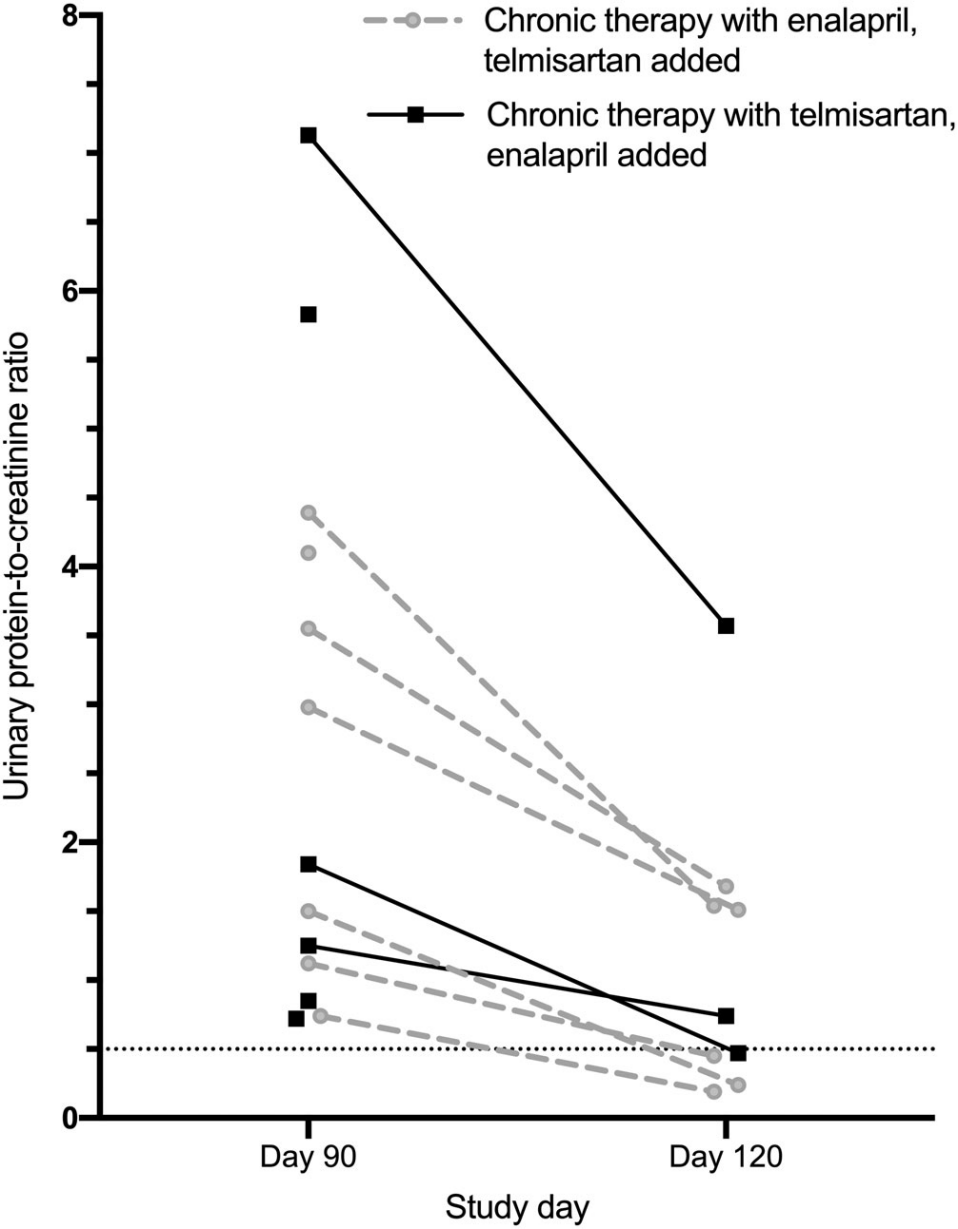
Urinary protein‐to‐creatinine ratio (UPC) before and after 30 days of combination therapy with enalapril and telmisartan in dogs with UPC > 0.5 at “ceiling” dosages of either medication alone. Dogs previously treated with enalapril (final dosage, 1.5 mg/kg PO q12h) had telmisartan (1 mg/kg PO q24h) added (gray circles). Dogs previously treated with telmisartan (final dosage, 3 mg/kg PO q24h) had enalapril (0.5 mg/kg PO q12h) added (closed squares). Lines connect measurements from a given individual. After addition of the second drug and prior to day 120, 4 dogs were removed from the study for percentage increase in blood creatinine >30% relative to baseline

Combination therapy was tested in 13 dogs (n = 7 enalapril‐treated, n = 6 telmisartan‐treated). Of these, 4 (31%; n = 3 in which enalapril, and n = 1 in which telmisartan was added) were removed from the study at day 97 for Δ_%_Cr >30%. UPC was lower at day 120 compared to day 90 in all 9 of the remaining dogs, of which 4 (n = 1 in which enalapril, and n = 3 in which telmisartan, was added) had UPC ≤0.5 at day 120 (Figure 7). Mean percentage change in UPC after 1 month of combination therapy (ie, percentage change in UPC at day 120 relative to day 90), was not significantly different between treatment groups (−55 ± 17% for telmisartan‐treated dogs in which enalapril was added vs −64 ± 13% for enalapril‐treated dogs in which telmisartan was added; *P* = .48).

**FIGURE 8:**
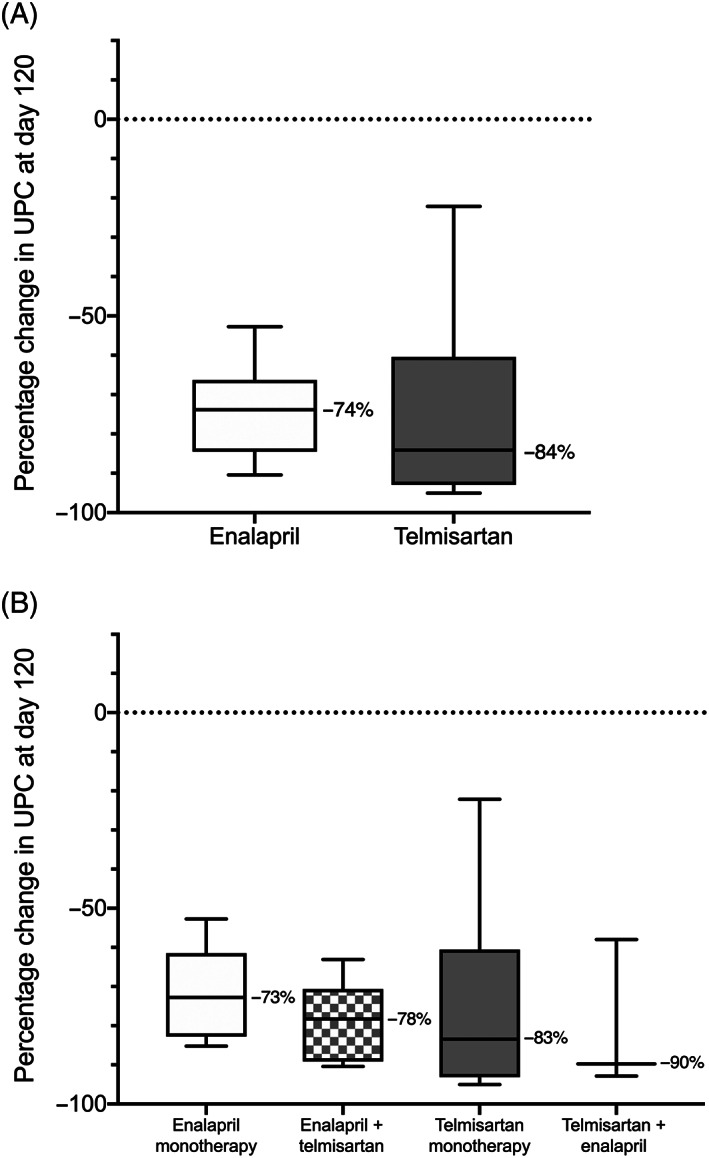
Box‐plot of change in urinary protein‐to‐creatinine ratio after 120 days of therapy in proteinuric dogs initially randomized to receive enalapril (n = 12) or telmisartan (n = 13). A, Data from all dogs of each study arm (ie, dogs receiving monotherapy and dogs receiving combination therapy), grouped according to identity of the initial study drug. B, Data from dogs grouped according study drug protocol administered for the 30 days preceding final study recheck (ie, enalapril monotherapy [0.5‐1.5 mg/kg PO q12h; n = 6], telmisartan monotherapy [1‐3 mg/kg PO q24h; n = 10], the combination of 1.5 mg/kg enalapril PO q12h + 1.0 mg/kg telmisartan q24h [n = 6], or the combination of 3 mg/kg telmisartan q24h + 0.5 mg/kg enalapril PO q12h [n = 3]). Boxes represent interquartile range, and the horizontal bar within each box and numbers to the right of it represent the median. Upper and lower bars are plotted using the method of Tukey

All 25 dogs that completed the 120‐day study period had a reduction in UPC relative to baseline value at day 120 (Figure 8). When data from all dogs that had started the same study drug (regardless of later addition of the other drug) were considered together, there were no significant differences in median Δ_%_UPC at day 120, and the proportion of dogs or odds of achieving UPC reduction from baseline ≥50% (Table 4) or UPC ≤0.5 (Table 5) at day 120 between treatment groups.

### 3.5 Time‐to‐event analyses

**FIGURE 9:**
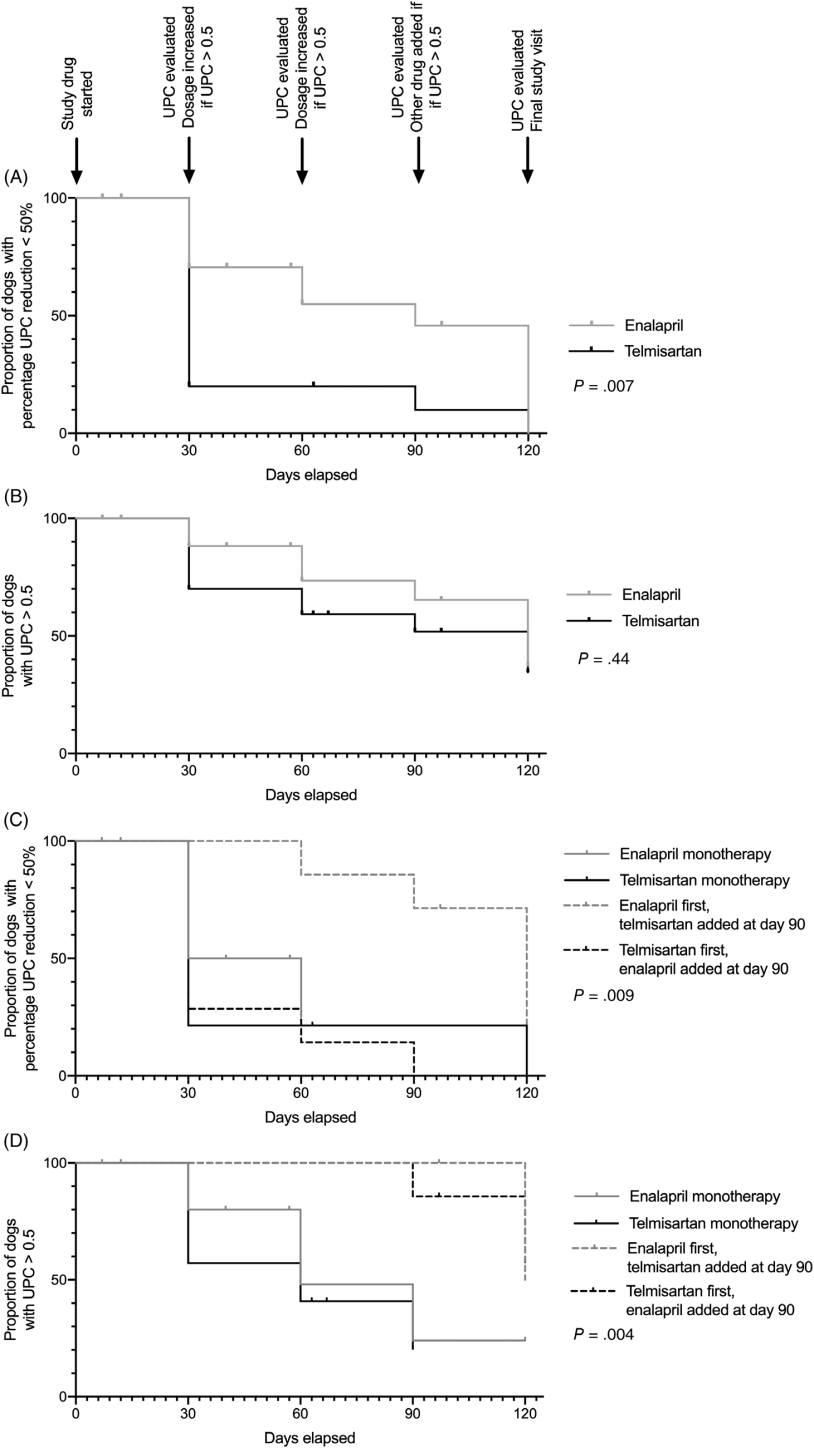
Kaplan Meier curves plotting the proportion of dogs in each treatment group that had not met the clinical endpoint of urinary‐protein‐to‐creatinine ratio (UPC) reduction ≥50% (A and C), or UPC ≤0.5 (B and D) over time. Dogs were treated with enalapril (0.5‐1.5 mg/kg PO q12h) or telmisartan (1‐3 mg/kg PO q24h) monotherapy during study days 0‐90, with or without the second drug during study days 91‐120. UPC was measured every 30 days until UPC ≤0.5 was achieved, and study drug dosages were increased every 30 days to target UPC ≤0.5. At day 90, dogs with UPC > 0.5 on “ceiling” dosages of either medication alone were treated with combination therapy. Vertical hashes represent dogs censored from analysis. A and B, Data from dogs grouped according to identity of the initial study drug. C and D, Data from dogs grouped according to study drug protocol administered for the 30 days preceding final study recheck (ie, enalapril or telmisartan monotherapy, the combination of 1.5 mg/kg enalapril PO q12h + 1.0 mg/kg telmisartan PO q24h, or the combination of 3 mg/kg telmisartan PO q24h + 0.5 mg/kg enalapril PO q12h)

Median time‐to‐UPC reduction from baseline ≥50% was significantly shorter in telmisartan‐ vs enalapril‐treated dogs (30 and 90 days, respectively; *P* = .007; Figure 9). There was no significant difference in time‐to‐UPC ≤0.5 between study groups.

### 3.6 Systolic blood pressure

In dogs not treated with amlodipine, for which average baseline SBP was 150 ± 14 and 148 ± 17 mm Hg in the enalapril‐ and telmisartan‐treated groups, respectively, mean Δ_%_SBP reduction was significantly greater in telmisartan‐ as compared to enalapril‐treated dogs at each of days 30 ± 2, 60 ± 2, 67 ± 2, and 97 ± 2 (Table 6). Hypotension was not observed at any visit.

**TABLE 6:** Percentage change in systolic blood pressure relative to baseline in proteinuric dogs randomized to receive enalapril or telmisartan, with or without the other study drug after day 90, and no concurrent treatment with amlodipine

Study day	Enalapril group		Telmisartan group		*P*‐value
	Value	n	Value	n	
7	−0.2 ± 13.60	13	−4.9 ± 11.9	16	.35
30	−0.9 ± 11.7	12	−12.3 ± 9.3	16	.01
37	1.3 ± 14.1	10	−5.2 ± 9.1	10	.25
60	4.5 ± 9.8	9	−10.0 ± 8.6	10	.004
67	8.2 (−17.7‐14.8)	6	−9.2 (−20.8‐6.7)	6	.04
90	1.1 ± 12.4	6	−9.1 ± 13.1	6	.20
97	6.6 ± 10.6	4	−24.0 ± 10.7	4	.007
120 (all dogs)	−11.2 ± 11.3	8	−13.3 ± 14.6	13	.72
120 (monotherapy)	−13.9 ± 9.2	5	−9.9 ± 11.8	10	.49
120 (combination therapy)	−6.7 ± 15.1	3	−24.5 ± 20.0	3	.29

*Note*: Data are presented as mean ± SD or median (range) where appropriate.

### 3.7 Safety variables

No significant differences in Δ_%_Cr or Δ_%_K were observed between groups at any evaluated time point (Table 7). However, 5 dogs of each treatment group were removed for Δ_%_Cr >30% alone (9/10) or in combination with hyperkalemia (1/10). Two (10%) of 20 dogs treated with telmisartan alone and 4 (21%) of 19 dogs treated with enalapril alone experienced Δ_%_Cr >30%. Of these, 1 (telmisartan‐treated) dog had concurrent vomiting, diarrhea, bacteriuria, and a positive urine culture (*E. coli*, >100 000 cfu/mL), raising concern for possible pyelonephritis. Four (31%) of 13 dogs receiving combination therapy (n = 1 in which telmisartan, and n = 3 in which enalapril, was added) were removed for Δ_%_Cr >30%. In all cases, Cr returned to baseline after discontinuation of study drugs; however, 1 dog that was receiving 3 mg of telmisartan/kg q24h and 0.5 mg of enalapril/kg q12h required in‐hospital treatment.

**TABLE 7:** Percentage change relative to baseline in blood creatinine (Δ%Cr) and potassium (Δ%K) concentrations and hematocrit (Δ%Hct) in proteinuric dogs randomized to receive enalapril or telmisartan, with or without the other study drug after day 90

Variable	Study day	Enalapril group		Telmisartan group		*P*‐value
		Value	n	Value	n	
Δ%Cr	7	9.6 ± 19.8	19	3.0 ± 20.1	20	.32
	30	11.0 ± 20.4	17	2.7 ± 21.5	20	.24
	37	9.5 ± 27.1	14	10.1 ± 16.4	13	.95
	67	14.3 ± 26.3	9	21.5 ± 22.7	9	.54
	97	20.0 (5.9‐150.0)	7	77.8 (4.5‐120.5)	6	.56
	120 (all dogs)	15.6 (−23.1‐50.0)	12	14.3 (−11.1‐88.9)	13	.70
	120 (monotherapy)	13.1 ± 17.0	6	10.9 ± 16.6	10	.81
	120 (combination therapy)	23.7 ± 27.2	6	37.7 ± 49.1	3	.67
Δ%K	7	4.0 ± 9.7	19	3.5 ± 9.2	20	.87
	30	4.5 (−7.2‐37.5)	17	3.3 (−9.5‐21.6)	20	.42
	37	5.0 ± 7.9	14	6.4 ± 5.3	13	.59
	67	1.8 (−3.7‐31.6)	9	6.2 (−8.0‐21.7)	9	.80
	97	13.8 ± 8.0	7	12.3 ± 9.8	6	.84
	120 (all dogs)	8.7 ± 11.7	12	5.4 ± 6.7	13	.40
	120 (monotherapy)	2.0 ± 10.6	6	3.6 ± 6.2	10	.76
	120 (combination therapy)	15.5 ± 9.0	6	11.4 ± 5.4	3	.43
Δ%Hct	7	0.0 (−13.9‐25.7)	19	−1.0 (−15.4‐9.4)	20	.73
	30	0.0 (−13.3‐34.3)	17	−4.9 (−19.2‐6.5)	20	.06
	37	−4.3 (−16.7‐42.9)	14	−9.4 (−23.1‐6.7)	13	.08
	67	3.1 ± 15.4	9	−9.9 ± 10.1	9	.05
	97	5.1 ± 13.0	7	−12.1 ± 5.8	6	.01
	120 (all dogs)	−2.9 ± 11.1	12	−8.5 ± 11.9	13	.25
	120 (monotherapy)	−6.8 ± 7.0	5^a^	−5.4 ± 11.4	10	.78
	120 (combination therapy)	0.3 ± 13.4	6	−18.7 ± 7.6	3	.03

*Note*: Data are presented as mean ± SD or median (range) where appropriate.

^a^ For 1 dog, hematocrit was not reported in the whole blood renal biochemical analysis performed at day 120.

While no significant differences in Δ_%_Hct were observed between groups at earlier time points, telmisartan‐treated dogs in which enalapril was added had significantly greater Δ_%_Hct reduction 1 week (ie, on study day 97 ± 2) and 1 month (ie, on study day 120 ± 2) after starting combination therapy than did those receiving the opposite combination.

### 3.8 Adverse events

Owner‐reported adverse events are summarized in Table 8. With the exception of 2 dogs which developed lethargy and anorexia after starting combination therapy, reported events were considered mild, and none was itself a cause for early removal from the study.

**TABLE 8:** Summary of owner‐reported adverse events from telmisartan‐treated (n = 20) and enalapril‐treated (n = 19) dogs

Adverse events		Enalapril monotherapy			Telmisartan monotherapy			Combination therapy	
		0.5 mg/kg q12h	1.0 mg/kg q12h	1.5 mg/kg q12h	1 mg/kg q24h	2 mg/kg q24h	3 mg/kg q24h	Enalapril, 1.5 mg/kg q12h + telmisartan, 1 mg/kg q24h	Telmisartan, 3 mg/kg q24h + enalapril 0.5 mg/kg q12h
Activity	Lethargy/decreased activity	2	–	–	2	1	–	–	1
	Somnolence	1	–	–	1	–	–	–	–
	Irritability	–	–	1	–	–	–	–	–
Appetite	Inappetence	6	1	2	4	4	2	2	5
	Increased appetite	1	2	–	–	–	–	–	–
Gastro‐intestinal	Vomiting	3	1	2	4	1	2	1	–
	Diarrhea	2	1	–	6	2	2	1	–
Urinary	Polyuria/Polydipsia	2	–	1	–	–	–	–	–
	Pollakiuria	1	–	–	–	–	–	–	–
	Urinary incontinence	1	2	–	1	–	–	–	–
	Nocturia	–	–	–	1	–	–	–	–
	Pigmenturia	–	–	–	–	–	1	–	–
	Decreased urination	–	–	–	1	–	–	–	–
	Urinary tract infection	–	–	–	1	–	–	–	–
Respiratory	Cough	1	–	–	–	1	–	–	–
	Reverse sneezing	1	–	–	–	–	–	–	–
Dermatologic	Pruritus	–	1	–	–	–	–	–	–
	Aural hematoma	–	1	–	–	–	–	–	–
	Acute moist dermatitis	–	–	–	1	–	–	–	–
Other	Corneal ulcer	–	–	1	–	–	–	–	–
	Tooth root abscess	–	1	–	–	–	–	–	–
	Ataxia	1	–	–	–	–	–	–	–
	Lameness	–	–	–	–	1	–	–	–
	Vestibular disease	–	–	–	1	–	–	–	–

*Note*: Data are presented as number of dogs for which each event was reported.

## 4 DISCUSSION

In the present study, treatment with telmisartan at a dosage of 1 mg/kg PO q24h led to significantly greater median reduction in UPC and a greater proportion of dogs with UPC reduction from baseline ≥50% after 30 days, compared to treatment with 0.5 mg enalapril/kg PO q12h. At progressively higher dosages of each drug, telmisartan's antiproteinuric effects were superior to those of enalapril, although only data from dogs refractory to lower dosages were included in these comparisons. Further, treatment with telmisartan resulted in UPC reduction in all but 1 dog (which was later found have active *B. burgdorferi* infection) in which it was administered, while 4 (24%) of 17 dogs treated with enalapril alone experienced an increase in UPC.

A previous prospective, masked, placebo‐controlled clinical trial of dogs with naturally‐occurring proteinuria demonstrated clinically relevant improvement (ie, UPC reduction ≥50% with stable serum Cr) in 9 (56%) of 16 dogs treated with 0.5 mg enalapril/kg q12‐24 hours for 6 months, and in no dogs treated with placebo for 6 months.^17^ In the present study, UPC reduction ≥50% was observed in 35.3% of enalapril‐treated and 80% of telmisartan‐treated dogs after 30 days of therapy.

In a preclinical study of healthy dogs, treatment with 1 mg telmisartan/kg PO q24h attenuated the systolic pressor response to exogenous Ang I by a significantly greater degree than placebo, enalapril (0.5 mg/kg q12h), and losartan (Coleman AE, Schmiedt CW, Handsford CG, et al. Attenuation of the pressor response to exogenous angiotensin by angiotensin receptor blockers in normal dogs. Journal of Veterinary Internal Medicine 2014;28:1002 [abstract]). These data, in addition to those of the present clinical trial, suggest that telmisartan might provide more clinically relevant RAAS blockade, and therefore, superior antiproteinuric effects, compared to enalapril. Similar observations have been made in human subjects, for whom a meta‐analysis of 20 randomized, controlled clinical trials including >25 000 people favored telmisartan over placebo, ACEi, other ARBs, non‐RAAS‐blocking antihypertensive drug therapy, or no medication, for the improvement of proteinuria or albuminuria.^38^ While 1 veterinary case report has described the successful use of telmisartan for the treatment of a dog with proteinuria that was refractory to benazepril therapy,^39^ systematic evidence regarding the efficacy of any ARB for the treatment of proteinuria in dogs has been lacking to date.

When the present study was designed, the use of combination ACEi/ARB therapy was relatively common in human medicine and associated with beneficial hemodynamic effects.^40^, ^41^, ^42^, ^43^, ^44^ Therefore, we also sought to evaluate the efficacy of this combination in dogs with persistent proteinuria despite treatment with “ceiling dosages” of enalapril or telmisartan. More recently, studies evaluating dual RAAS inhibition in humans report failure to improve cardiovascular or renal outcomes and increased risk of adverse events, despite improved efficacy compared to monotherapy.^45^, ^46^, ^47^ Consequently, combination ACEi/ARB therapy is no longer a general recommendation for treatment of human renal or cardiovascular diseases. The findings of the present study raise similar concerns for dogs. While dual RAAS blockade led to UPC reduction and UPC ≤0.5 in 100% and 44% of dogs in which it was tolerated, respectively, 31% of dogs in which it was tested developed clinically relevant increases in Cr within 7 days, with 1 (IRIS stage 1 at baseline) requiring hospitalization for treatment of suspected acute kidney injury.^48^ The authors therefore advise caution when combining these medications, although it deserves comment that dual therapy was performed by combining maximum tested dosages of 1 RAAS blocker with a starting dosage of the other. In 1 study, the combination of candesartan and ramipril at low dosages of each was safe and efficacious for proteinuria reduction in human patients with advanced CKD.^42^ Whether the same would be true for dogs remains to be studied.

Azotemia, hyperkalemia, and acute kidney injury are well‐described potential adverse effects of RAAS blockade in patients with renal or cardiac disease.^49^ Excluding those dogs in which combination therapy was tested, 6 dogs (n = 4 enalapril‐treated and n = 2 telmisartan treated) were removed from the present study prior to day 120 due to Δ_%_Cr >30% and in 1 case, substantial hyperkalemia. Of these, 2 dogs (both enalapril‐treated) were receiving the starting dosage at the time of removal, and the remainder were receiving higher study drug dosages. There were no significant differences in Δ_%_Cr or Δ_%_K between treatment groups at any evaluated timepoint.

The present study was neither specifically designed nor adequately powered to assess the relative effects of telmisartan and enalapril on SBP. Nonetheless, excluding dogs receiving amlodipine (and therefore, those with SBP ≥180 mm Hg), there was a significantly greater reduction in SBP in telmisartan‐, vs enalapril‐treated dogs at several study timepoints as early as study day 30. Clinical data regarding telmisartan's antihypertensive efficacy in dogs is limited to that of a single case series of 5 dogs with refractory systemic hypertension.^50^ Prospective research in spontaneously hypertensive dogs is warranted to explore this drug's antihypertensive efficacy in this species.

Ang II modulates erythropoeiesis, as its signaling regulates renal transcription of erythropoietin to increase proliferation of early erythroid progenitors.^51^, ^52^, ^53^ In the present study, although differences observed during the monotherapy phases did not reach statistical significance, dogs treated with “ceiling” dosages of telmisartan for which enalapril was added had a greater decrease in mean Hct over the month of combination therapy than did those treated with the opposite combination. Observed changes in Hct were uncommonly clinically relevant. Nonetheless, further study, including the measurement of serum erythropoietin concentrations in treated dogs, might help to clarify the impact of RAAS blockers on red blood cell homeostasis in dogs.

There are several limitations to this study. Because renal biopsies were not performed, we are unable to describe our sample in terms of specific histologic diagnoses. However, treatment randomization makes it unlikely that dogs with specific conditions (eg, immune‐complex glomerulonephritis^54^) that could be less responsive to RAAS inhibition than others, were disproportionally assigned to a particular treatment group. A second major limitation is the lack of a complete set of monthly recheck data for all dogs which reached study end. Because regular rechecks were performed only until the clinical goal of UPC ≤0.5 was reached (after which the dog was not reevaluated until day 120 ± 2), sets from scheduled visits between study day 30 ± 2 and 120 ± 2 do not include data from dogs that met this goal at an earlier timepoint. Therefore, comparisons of median UPC reduction at days 60 and 90 are biased toward “nonresponders,” and proportions of dogs achieving a given clinical goal at these timepoints only take into account dogs actually rechecked. Further, the number of dogs evaluated at these time points is relatively small, impacting the power to detect treatment group differences at those visits. A final limitation is that a now‐commercially available formulation of telmisartan was compared to a compounded formulation of enalapril. However, as described in the methods, compounding of enalapril suspension from tablets is a practice endorsed by the United States Pharmacopeia,^32^ and several measures were taken to ensure adequate quality and to minimize product variability of the enalapril suspension tested. Product stability testing of the enalapril suspension used in the present study was not performed. Therefore, the quality of the product cannot be confirmed. Nonetheless, there was evidence of pharmacodynamic responses in the dogs receiving the enalapril suspension alone and in combination with telmisartan.

In conclusion, after 30 days of therapy using the dosages and formulations tested, telmisartan treatment led to a greater percentage reduction in UPC than did enalapril treatment, and telmisartan treatment produced a clinically relevant reduction in UPC in a greater proportion of dogs and a shorter period of time. For dogs remaining proteinuric while receiving standard dosages of these medications, the antiproteinuric effects of increasing dosages of telmisartan were superior to those of similarly increasing dosages of enalapril; however, these data are limited by the smaller number of dogs evaluated at the later phases of the present study. No clear differences in the safety profiles of these medications, when administered alone, were observed. These data suggest that telmisartan is a suitable first‐line choice for RAAS inhibition in dogs with renal proteinuria.

## CONFLICT OF INTEREST DECLARATION

Dr Bianca Lourenço was the recipient of a Boehringer Ingelheim Postdoctoral Scholarship. Drs Amanda Coleman and Scott Brown have served as paid consultants for Boehringer Ingelheim Vetmedica GmbH.

## OFF‐LABEL ANTIMICROBIAL DECLARATION

Authors declare no off‐label use of antimicrobials.

## INSTITUTIONAL ANIMAL CARE AND USE COMMITTEE (IACUC) OR OTHER APPROVAL DECLARATION

Approved by the Clinical Research Committee of the University of Georgia's College of Veterinary Medicine, approval number CR‐399.

## HUMAN ETHICS APPROVAL DECLARATION

Authors declare human ethics approval was not needed for this study.
